# Mesenteric adipose tissue B lymphocytes promote local and hepatic inflammation in non‐alcoholic fatty liver disease mice

**DOI:** 10.1111/jcmm.14232

**Published:** 2019-02-17

**Authors:** Zhe Wu, Jun Xu, Jiang Tan, Yang Song, Ling Liu, Feng Zhang, Yifan Zhang, Xia Li, Yujing Chi, Yulan Liu

**Affiliations:** ^1^ Department of Gastroenterology Peking University People's Hospital Beijing P.R. China; ^2^ Clinical Center of Immune‐Mediated Digestive Diseases Peking University People's Hospital Beijing P.R. China; ^3^ Gerontology Peking University People's Hospital Beijing P.R. China; ^4^ Department of Central Laboratory & Institute of Clinical Molecular Biology Peking University People's Hospital Beijing P.R. China

**Keywords:** B lymphocytes, inflammation, macrophages, mesenteric adipose tissue, non‐alcoholic fatty liver disease

## Abstract

Mesenteric adipose tissue (MAT) inflammation is associated with non‐alcoholic fatty liver disease (NAFLD), and immune cells play pivotal roles in the inflammation of adipose tissue. Here, we investigated the roles of MAT B lymphocytes in NAFLD. Mice fed with high‐fat diet (HFD) and normal diet (ND) were killed in time gradients (4, 8 and 12 weeks). Compared with ND‐fed mice, intra‐hepatic CD45^+^CD19^+^ B lymphocytes increased after 4 weeks (*P* < 0.01) of HFD feeding, and lasted until the 12th week, infiltrated earlier than CD45^+^CD3^+^ T lymphocytes and CD45^+^F4/80^+^ macrophages. The mRNA expression of tumour necrosis factor (TNF)‐α, interleukin (IL)‐6 and monocyte chemotactic protein (MCP)‐1 decreased in MAT of B^null^ HFD‐fed mice compared to that in wild‐type HFD‐fed mice, along with lesser macrophages. Mesenteric adipose tissue B cells from HFD‐fed mice promoted macrophage differentiation to type‐Ι macrophages and expression of pro‐inflammatory cytokines in vitro. Macrophages pre‐treated with MAT B cells from HFD‐fed mice showed elevated mRNA expression of IL‐6 and TNF‐α and declined IL‐10 levels in adipocytes compared to ND MAT B cell pre‐treated macrophages. Besides, internal near‐infrared scanning and external transwell assay showed that HFD MAT B cells migrated to the liver more than ND MAT B cells. High‐fat diet MAT B cells induced higher MCP‐1 and lower IL‐10 expression in primary hepatocytes compared to ND MAT B cells in co‐culture experiment. These data indicate that B lymphocytes infiltrate early in MAT during the development of NAFLD, which may not only promote MAT inflammation by regulating macrophages but also migrate to the liver and induce hepatocytes inflammation.

## 1 INTRODUCTION

Non‐alcoholic fatty liver disease (NAFLD) has become the most common chronic liver disease worldwide and is an independent risk factor for type 2 diabetes mellitus (T2DM), cardiovascular disease, etc.^1^, ^2^ Visceral adipose tissue (VAT) is implicated in the development of NAFLD.^3^, ^4^ As a depot of VAT, mesenteric adipose tissue (MAT) is located between the gut and liver, which makes it possible to affect the liver through the portal vein by secreting pro‐inflammatory cytokines, adipocytokines and releasing free fatty acid (FFA), etc.^5^, ^6^ Mesenteric adipose tissue is now thought to play a more important role in the pathogenesis of obesity‐related liver diseases or in insulin resistance than other depots of VAT.^8^, ^9^ Rytka et  al found that mice receiving a portal drained fat transplant, similar to MAT, had worse hepatic insulin resistance compared to mice receiving vena cava drained fat.^6^ Intervention strategies targeting MAT have demonstrated that MAT can affect hepatic metabolism, specifically inhibiting MAT inflammation and resulting in less lipolysis and better hepatic insulin resistance and steatosis.^10^

Mesenteric adipose tissue inflammation contributes to NAFLD, but what promotes MAT inflammation is still not clear. In obesity‐related diseases, various kinds of lymphocytes such as macrophages, natural killer (NK) cells and CD8^+^ T cells are infiltrated and activated in MAT,^11^, ^12^ promoting inflammation and lipolysis and eventually aggravating metabolic disorders.^13^ In recent years, increasing studies have suggested that B lymphocytes are also involved in adipose tissue inflammation. B lymphocytes in epididymal adipose tissue (EAT) can promote EAT inflammation through modulation of T lymphocytes and production of immunoglobulin G (IgG) antibody in HFD‐fed mice.^14^ Transferred B2 cells in EAT of HFD‐fed mice to HFD‐fed B^null^ recipient mice result in increased adipose tissue inflammation.^15^ B^null ^mice show improved insulin resistance and glucose metabolism compared to control mice under the stimulation of HFD,^14^, ^15^ and B2 cell depletion in mice resulted in alleviated NAFLD.^16^ However, the role of B lymphocytes in the inflammation of MAT is poorly understood. Here, we aimed to explore the role of MAT B cells in MAT inflammation.

Recently, immune dialogue between the gut and liver has drawn researchers’ attention. Studies have shown the existence of enterohepatic lymphocyte circulation.^17^ In our previous study, we proved that immune cells including T and B lymphocytes could shuttle from mesenteric lymph nodes to the liver and promote NAFLD.^18^ As mentioned above, MAT is rich in lymphatic and blood vessels draining in the portal vein, laying the structural and physiological foundation of lymphocyte circulation between MAT and the liver. In the current study, we also investigated whether MAT B cells could migrate to the liver and their effects on NAFLD.

Our results showed that during the development of NAFLD, B lymphocytes infiltrated earlier than macrophages in MAT and might promote MAT inflammation through modulation of macrophages. Besides, the migration of MAT B lymphocytes to the liver increased, promoting hepatic inflammation in NAFLD.

## 2 MATERIALS AND METHODS

### 2.1 Animals and NAFLD model

Six‐to‐eight‐week‐old male C57BL/6J wild‐type and B6.129S2 muMT mice (homozygous mutant mice that lack mature B cells, B^null^ mice) were purchased from Beijing Vital River Laboratory Animal Technology Co., Ltd. (Beijing, China) and Jackson Laboratory (Bar Harbor, ME, USA) respectively. Mice were raised in a specific pathogen‐free (SPF) facility at Peking University People's Hospital. Thirty‐six wild‐type male mice were randomly divided into six groups. Half of them were fed with 45% kcal high‐fat diet (HFD) (Mediscience Ltd., Yangzhou, China), and the others with normal diet (ND). Six mice from each group were killed in time gradients (4, 8 and 12 weeks of feeding). Six 6‐8‐week‐old muMT mice and control mice were killed after 12 weeks of HFD feeding. All protocols were approved by the Peking University People's Hospital Ethics Committee.

### 2.2 Isolation of lymphocytes in the liver and adipose tissue

Liver was perfused with PBS via the portal vein to eliminate the effects of lymphocytes in blood. Single‐cell suspensions were prepared by mechanically disrupting liver and went through a 100‐µm filter. Intrahepatic lymphocytes were isolated using 40% and 80% discontinuous Percoll (GE healthcare Bio‐Sciences AB, Uppsala, Sweden) density gradient centrifugation. The cells in the middle layer were collected and washed with PBS three times.

Fresh adipose tissues were cut into pieces and digested with 1 mg/mL type‐Ι collagenase (Sigma‐Aldrich, Merck Life Science (Shanghai) Co., Ltd. Shanghai, China) and 0.4 g/mL bovine serum albumin (BSA) dissolved in PBS for 30 minutes at 37°C with shaking at 200 rpm. Undigested impurities were excluded using a 70‐µm strainer, and soluble supernatants were centrifuged for 10 minutes at 300× *g *to acquire precipitated stromal vascular fractions (SVFs) that were used for flow cytometry after washing with PBS three times.

### 2.3 Flow cytometry

Lymphocytes were labelled with CD45‐PE, CD19‐APC, CD3‐FITC and F4/80‐PerCP/Cy5.5 antibodies (Biolegend, San Diego, CA, USA) and detected by a FACS Calibur flow cytometer (BD Immunocytometry Systems, Franklin Lakes, NJ). The data were analysed using Flowjo 7.6 (Flowjo, Ashland, OR).

### 2.4 Immunofluorescence staining and confocal imaging assay

Adipose tissues were fixed in optimal cutting temperature (OCT) (Sakura Finetek USA, Torrance, CA) and cut at 10‐µm thickness at −30°C using a Leica frozen slicer. The slides of the tissues were blocked with 1% BSA for 30 minutes, followed by 1‐hour incubation with anti‐CD19 antibody (1:200; Abcam, Shanghai, China) and anti‐F4/80 antibody (1:200; Abcam) at 37°C. Slides were incubated for 30 minutes with goat anti‐rat IgG Alexa Fluor 488 and goat anti‐mouse IgG Alexa Fluor 594 (1:200; Invitrogen, Rockford, IL). 4′,6‐diamidino‐2‐2phenylindole (DAPI) (Cell Signaling Technology, Danvers, MA) was used to stain the nuclei for 2 minutes after the slides were thoroughly washed. The slides were observed using a confocal microscope (FV 1000; Olympus, Tokyo, Japan).

### 2.5 B lymphocyte and primary hepatocyte isolation and cultivation

CD19^+^ B cells in SVFs were sorted using MACS (Miltenyi Biotec, Bergisch Gladbach, Germany) following the manufacturer instructions. B cells were cultured in Roswell Park Memorial Institute (RPMI) 1640 complete medium supplemented with 10% foetal bovine serum (FBS) (Gibco, Gaithersburg, MD, USA) and 1% 100× penicillin/streptomycin.

Primary hepatocytes from mice were isolated using the two‐step perfusion method, as described by Madsen et  al.^19^ Hepatocytes (2 × 10^5 ^cells/mL) were plated in a 6‐well plates coated with rat tail collagen, with DMEM (glucose 4500 mg/L, L‐glutamine and 110 mg/L sodium pyruvate; Gibco) supplemented with 10% FBS, 1% 100× penicillin/streptomycin and 10 nmol/L 4‐(2‐hydroxyethyl)‐1‐piperazineethanesulfonic acid (HEPES).

### 2.6 B lymphocyte and macrophage co‐culture experiments

RAW264.7 macrophages (2 × 10^5 ^cells/mL) were cultured in RPMI 1640 complete medium, and an equal number of B lymphocytes from MAT was co‐cultured in a transwell plate for 24 hours, and they were separated by a 0.4‐μm track‐etched membrane. Macrophages were collected for RNA extraction and cDNA synthesis.

In another experiment, the co‐culture system of RAW264.7 macrophages and MAT B lymphocytes was added to 500 ng/mL interferon γ (IFN‐γ) and 1 µg/mL lipopolysaccharide (LPS). After 24 hours of cultivation, RAW264.7 cells were collected and labelled with F4/80‐PerCP/Cy5.5, CD11c‐APC and CD206‐FITC antibodies and detected using a FACS Calibur flow cytometer.

### 2.7 Macrophage and adipocyte co‐culture experiments

3T3‐L1 cells were cultured in DMEM with 10% FBS and differentiated into mature adipocytes using insulin, dexamethasone and 3‐isobutyl‐1‐methylxanthine. Mature adipocytes (2 × 10^5^ cells/mL) were cultured with an equal number of RAW264.7 macrophages for 24 hours in the co‐culture system, in which macrophages were pre‐treated with an equal number of ND and HFD B cells or were not pre‐treated for 24 hours. Adipocytes were harvested for RNA extraction.

### 2.8 Immunohistochemistry

Mesenteric adipose tissue was fixed, dehydrated and embedded in paraffin wax. Sections (4‐µm thick) were incubated with anti‐F4/80 antibody (1:200) or IgG at 4°C overnight. After incubation with a poly‐peroxidase‐conjugated goat anti‐rat IgG (Zhongshan Golden Bridge, Beijing, China) at 37°C for 30 minutes, the slides were counterstained with 3,3′‐diaminobenzidine (DAB) and haematoxylin and eventually mounted with mounting medium (DPX). The number of F4/80^+^ macrophages was quantitated in three randomly selected high‐power fields (HPFs) 200× of each slide.

### 2.9 1,1′‐Dioctadecyl‐3,3,3′,3′‐tetramethylindotricarbocyanine iodide‐labelled B lymphocyte transfusion for near‐infrared scanning in vivo

B lymphocytes within MAT were isolated from 12‐week HFD‐ and ND‐fed mice as described above. B lymphocytes were incubated with 20 µmol/L 1,1′‐dioctadecyl‐3,3,3′,3′‐tetramethylindotricarbocyanine iodide (DiR) buffer (Molecular Probes; Thermo Fisher Scientific, Waltham, MA) for 15 minutes at 4°C, followed by washing with PBS three times. DiR‐labelled B lymphocytes (1 × 10^6^) in 0.2 mL of PBS or PBS alone were intravenously injected into 12‐week HFD‐ or ND‐fed mice. Recipient mice were anaesthetized using 2% isoflurane, and their abdomen was depilated. Near‐infrared scanning was performed with IVIS Imaging System (Caliper Life Sciences, Hopkinton, MA). Recipient mice were imaged at 2, 12 and 24 hours post‐injection. Pictures were analysed using Living Image software, version 4.2 (PerkinElmer, Waltham, MA).

### 2.10 Liver homogenate and transwell assay

Liver sample (100 mg) was homogenized and diluted in 5 mL RPMI 1640 medium to a 20% concentration. All the procedures were performed on ice. Cell debris was removed by centrifugation, and supernatant homogenates were stored. Afterwards, 0.2 mL 20% liver homogenates and 0.4 mL RPMI 1640 medium were added to the lower chamber of the transwell system (24‐well plates; 5.0 µm pore size; Costar, Corning), and RPMI 1640 medium (0.2 mL) with 10^5^ B cells was added to the upper chamber. After culture at 37°C for 6 hours, the number of B cells in the lower chamber was counted, and chemotactic index was calculated. Three wells per group were set, and the experiment was repeated three times.

### 2.11 B lymphocyte and primary hepatocyte co‐culture experiments

Primary hepatocytes (2 × 10^5^ cells/mL), either from ND‐ or HFD‐fed mice, were cultured with an equal number of MAT B cells for 24 hours in the same co‐culture system. Hepatocytes and cultural supernatant were harvested.

### 2.12 RNA extraction, cDNA synthesis and real‐time polymerase chain reaction

Total RNA was extracted using RNeasy Plus Mini kit (QIAGEN Co., Ltd., Hilden, Germany), and cDNAs were synthesized using RevertAid First Strand cDNA Synthesis kit (Thermo Fisher Scientific, Vilnius, Lithuania). Real‐time polymerase chain reaction (PCR) was performed with a StepOne Plus Real‐Time PCR System (Applied Biosystems, Waltham, MA) using SYBR Premix Ex Taq (Toyobo, Kita‐ku, Osaka, Japan). The sequences of primers are listed in Table S1. The 2^−△△Ct^ method was used to compare relative mRNA expressions.

### 2.13 Enzyme‐linked immunosorbent assay

Levels of TNF‐α, MCP‐1 and IL‐10 in supernatants were measured using ELISA kits as per manufacturer's instruction (eBioscience, San Diego, CA).

### 2.14 Biochemistry detection

The concentrations of alanine aminotransferase (ALT) and aspartate aminotransferase (AST) were detected using an automatic biochemical detector‐Labospect 008 (Hitachi Ltd., Tokyo, Japan).

### 2.15 Statistical analysis

Data are presented as mean ± SEM. Two‐tailed student's *t* test or one‐way ANOVA were used to compare values between the groups. spss 20.0 (IBM, New York, NY, USA) was used to perform statistical analyses. *P < *0.05 was considered statistically significant.

## 3 RESULTS

### 3.1 B lymphocyte infiltrated early in the liver and MAT of HFD‐fed mice

**Figure 1:**
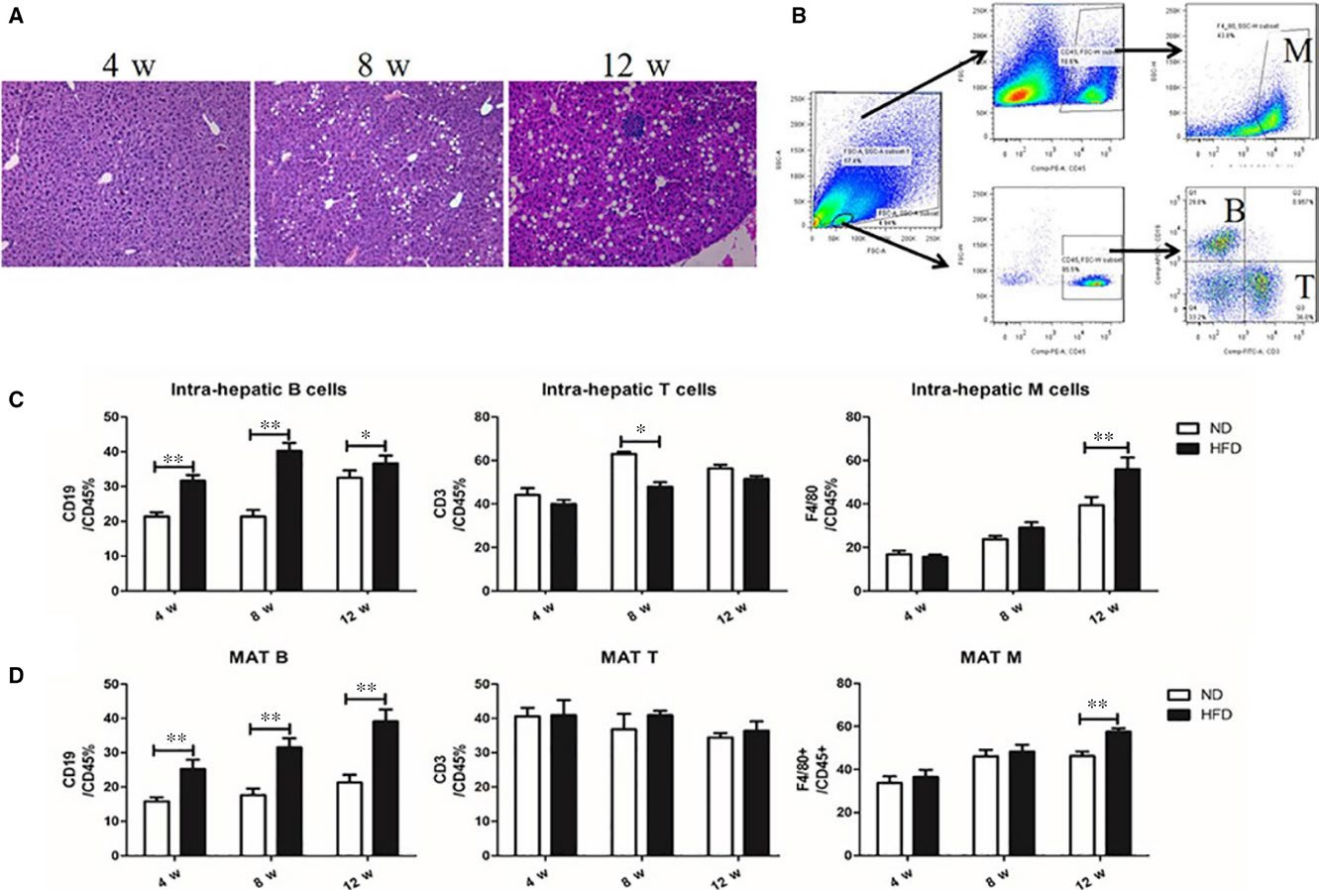
B lymphocyte infiltrated early in the liver and MAT of HFD‐fed mice. A, The representative liver sections of HFD‐fed mice for 4, 8 and 12 wk after haematoxylin and eosin staining, 100× magnification. B, The gating strategy of flow cytometry for the liver and MAT. The proportion of CD45^+^CD19^+^ B lymphocytes, CD45^+^CD3^+^ T lymphocytes and CD45^+^F4/80^+^ macrophages in the liver (C) and MAT (D) of HFD‐ and ND‐fed mice. Values represent means ± SEM, *P < 0.05, **P < 0.01. HFD, high‐fat diet; MΦ, macrophages; MAT, mesenteric adipose tissue; ND, normal diet

To investigate the role of immune cells in the MAT and liver of NAFLD mice, we detected them at multiple time points (4, 8 and 12 weeks) using flow cytometry. Haematoxylin and eosin staining showed that fat droplets increased in number and size in the livers of HFD‐fed mice as compared to that in control mice, and the number and size increased with the time period of HFD feeding (Figure 1A). The gating strategy of flow cytometry for the liver and MAT is shown in Figure 1B. CD45^+^CD19^+^ lymphocytes were considered as B cells, CD45^+^CD3^+^ lymphocytes were considered as T cells and CD45^+^F4/80^+^ lymphocytes were considered as macrophages. Compared with ND‐fed mice, intra‐hepatic CD45^+^CD19^+^ B lymphocytes increased after 4 weeks (*P* < 0.01) of HFD feeding, and lasted up to the 12th week (*P* < 0.05). CD45^+^CD3^+^ T lymphocytes decreased in the 8th week (*P* < 0.05) in HFD‐fed mice, and CD45^+^F4/80^+^ macrophages did not increase in HFD‐fed mice until the 12th week (Figure 1C). Similarly, B lymphocytes within MAT increased from the 4th week to the 12th week in HFD‐fed mice (*P* < 0.01). T lymphocytes showed no significant differences between the HFD‐fed and ND groups at three time points. Macrophages increased in the 12th week (*P* < 0.01) (Figure 1D). These data indicated that B lymphocytes infiltrated earlier than macrophages in HFD‐fed mice.

### 3.2 Macrophages and MAT inflammation decreased in B^null^ mice

**Figure 2:**
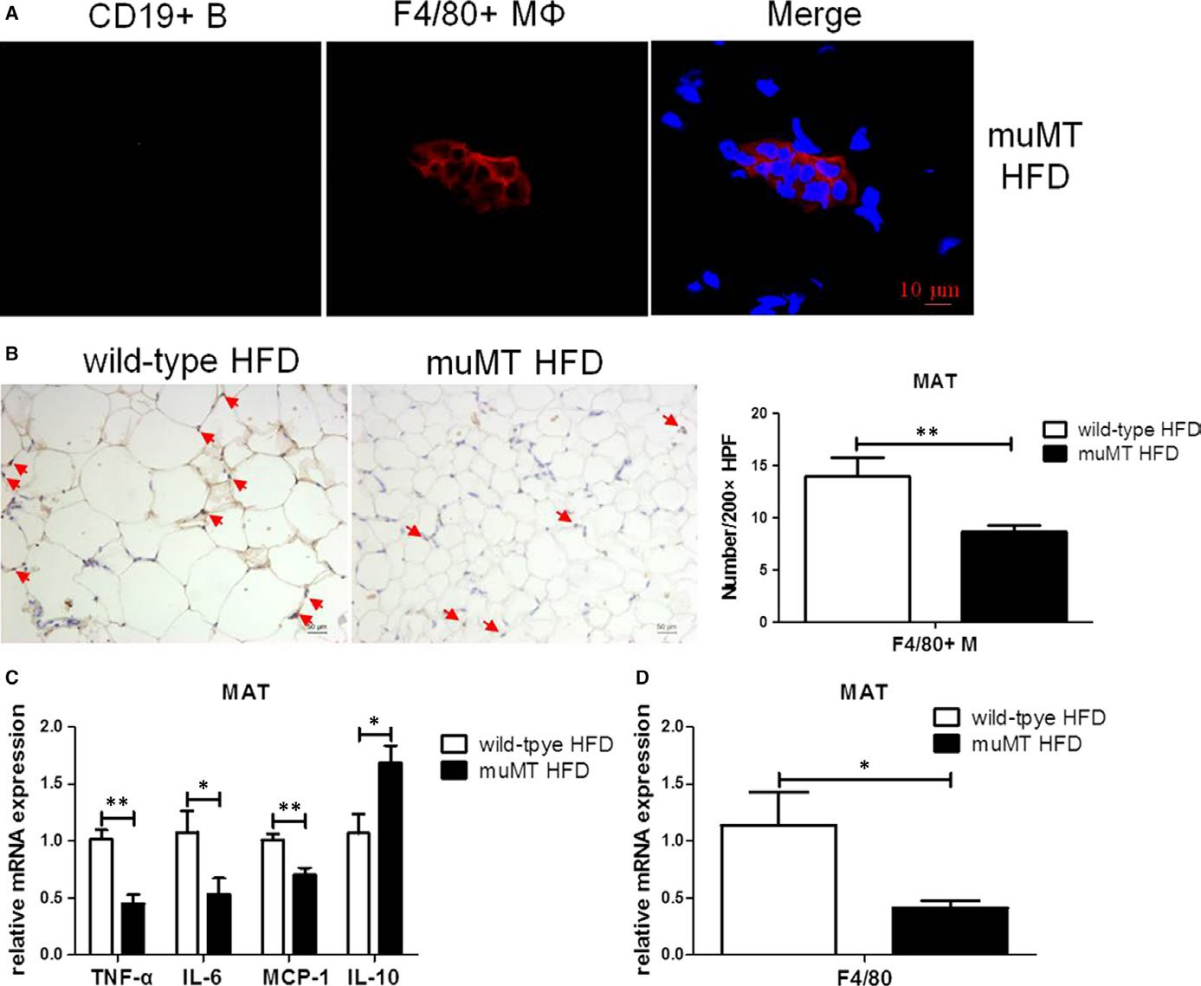
Macrophages and MAT inflammation decreased in B^null^ mice. A, The representative immunofluorescence staining of MAT of HFD‐fed muMT mice for 12 wk lacking mature B cells. The scale bar represents 10 μm. B, The representative immunohistochemistry staining (200× magnification) of MAT of HFD‐fed wild‐type and muMT mice; the scale bar represents 50 μm, and the red arrows point to F4/80 positive macrophages. Number of F4/80^+^ macrophages counted in high‐power fields (200×). The relative mRNA expression of F4/80 (C) and inflammatory cytokines (D) in MAT, specifically, TNF‐α, IL‐6, MCP‐1 and IL‐10. Values represent means ± SEM, *P < 0.05, **P < 0.01. HFD, high‐fat diet; IL, interleukin; MAT, mesenteric adipose tissue; TNF, tumour necrosis factor

muMT mice, lacking mature B lymphocytes, were used to confirm the role of B lymphocytes in MAT inflammation. Immunofluorescence staining evidenced the lack of B cells in HFD‐fed muMT mice (Figure 2A). Histological assessment showed that adipocytes were smaller in B^null^ mice than in wild‐type mice with HFD feeding (Figure 2B), and the mRNA expression of TNF‐α (*P* < 0.01), IL‐6 (*P* < 0.05) and MCP‐1 (*P* < 0.01) decreased, and that of IL‐10 (*P* < 0.05) increased in MAT of B^null^ mice (Figure 2C). Compared to HFD‐fed wild‐type mice, immunohistochemical staining showed lesser F4/80^+^ macrophages (*P* < 0.01) in MAT as well as decreased mRNA expression of F4/80 (*P* < 0.05) in HFD‐fed muMT mice (Figure 2B,D). These results implied that lack of B cells might partially contribute to the reduction of macrophages and alleviated MAT inflammation.

### 3.3 Infiltrated B lymphocytes might relate to macrophage inflammation and their differentiation into M1 macrophage

**Figure 3:**
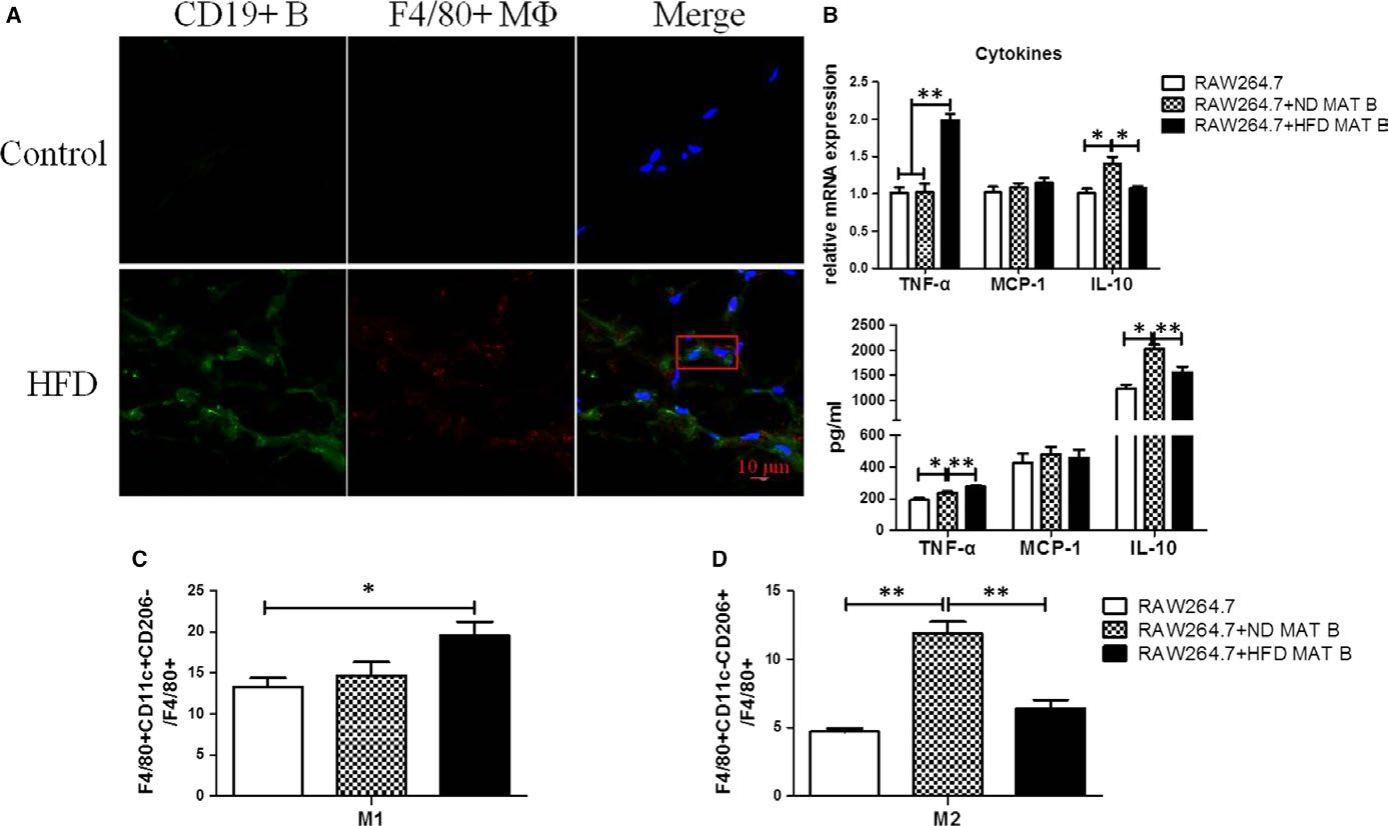
Infiltrated B lymphocytes might relate to macrophage inflammation and their differentiation into M1 macrophage. A, The representative immunofluorescence staining of MAT of HFD‐fed mice for 12 wk. The scale bar represents 10 μm. The red rectangle includes a macrophage and an adjacent B cell. B, The proportion of F4/80^+^CD11c^+^CD206^−^ M1 and F4/80^+^CD11c^−^CD206^+^ M2 macrophages in the co‐culture system of RAW264.7 macrophages and MAT B lymphocytes, either from normal diet (ND)‐ or HFD‐fed mice. C, The relative mRNA expression of inflammatory cytokines in macrophages of the co‐culture system, specifically, TNF‐α, MCP‐1 and IL‐10. D, The levels of cytokines in cultural supernatants of the RAW264.7 macrophages and MAT B cells co‐culture system, specifically, TNF‐α, MCP‐1 and IL‐10. Values represent means ± SEM, *P < 0.05, **P < 0.01. HFD, high‐fat diet; IL, interleukin; MAT, mesenteric adipose tissue; TNF, tumour necrosis factor

As mentioned above, B lymphocytes infiltrated earlier than macrophages in MAT. Besides, immunofluorescence staining showed that some B lymphocytes were located next to macrophages (Figure 3A). In the co‐culture experiment, MAT B cells of HFD‐fed mice (HFD MAT B cells) elevated the mRNA expression of TNF‐α (*P* < 0.01) and reduced the mRNA expression of IL‐10 (*P* < 0.05) in macrophages compared to MAT B cells of ND‐fed mice (ND MAT B cells) (Figure 3B). Levels of TNF‐α (*P* < 0.01) and IL‐10 (*P* < 0.01) in supernatants were consistent with the mRNA expression (Figure 3B). HFD MAT B cells promoted the differentiation of macrophages into F4/80^+^CD11c^+^CD206^−^ type I macrophages (*P* < 0.05) (Figure 3C). Normal diet MAT B cells promoted macrophage differentiation into F4/80^+^CD11c^−^CD206^+^ type II macrophages (*P* < 0.01) (Figure 3D), and HFD MAT B cells induced lesser type II macrophages than ND MAT B cell (*P* < 0.01) (Figure 3D). These evidences indicated that MAT B lymphocytes of HFD‐fed mice might contribute to macrophage inflammation, and relate to macrophage differentiation into M1 macrophages.

### 3.4 Infiltrated B lymphocytes might contribute to MAT inflammation by regulating macrophage

**Figure 4:**
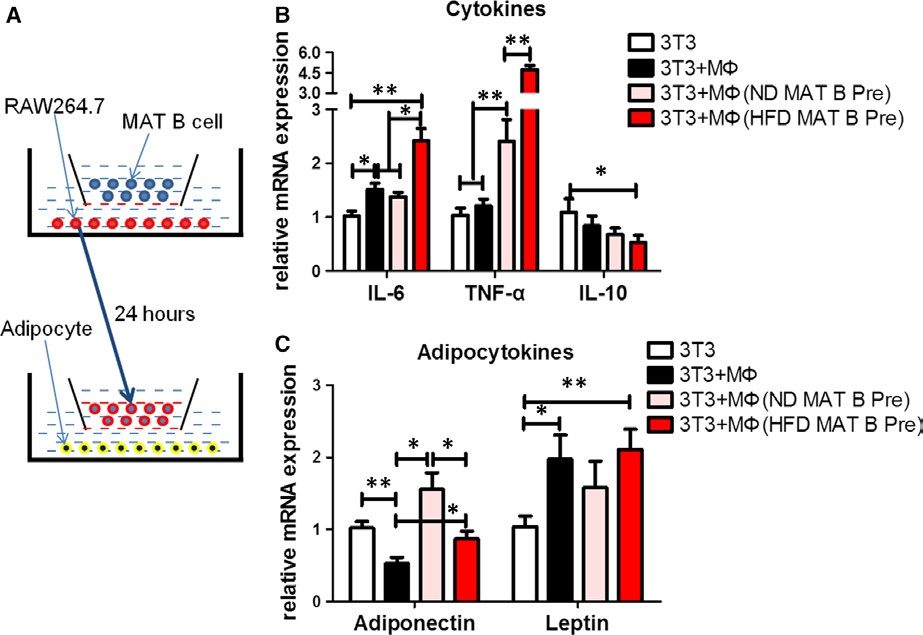
Infiltrated B lymphocytes might contribute to MAT inflammation by regulating macrophage. A, The schematic diagram of co‐culture system of mature adipocytes and RAW264.7 macrophages, with MAT B cells pre‐treated or not. Relative mRNA expression of adipocytokines (B) and inflammatory cytokines (C) of adipocytes, specifically, adiponectin, leptin, IL‐6, TNF‐α and IL‐10. Values represent means ± SEM, *P < 0.05, **P < 0.01. IL, interleukin; MAT, mesenteric adipose tissue; TNF, tumour necrosis factor

As far as we know, there was no B cell receptor on the surface of adipocytes; thus, we investigated whether B lymphocytes could affect adipocytes by regulating macrophages. The protocols of co‐culture experiments are shown in Figure 4A. Macrophages promoted mRNA expression of IL‐6 in mature adipocytes (*P* < 0.05), and TNF‐α or IL‐10 levels did not change (Figure 4B). The mRNA expression of leptin increased (*P* < 0.05) and that of adiponectin decreased (*P* < 0.01) after simple macrophage stimulation in mature adipocytes (Figure 4C). Compared with simple macrophages, increased mRNA level of TNF‐α (*P* < 0.01) and adiponectin (*P* < 0.05) in adipocytes were observed in macrophages pre‐treated with ND MAT B cells, as well increased IL‐6 (*P* < 0.05), TNF‐α (*P* < 0.01) and adiponectin (*P* < 0.05) in adipocytes in macrophages pre‐treated with HFD MAT B cells, and IL‐10 or leptin levels were not significantly different (Figure 4B,C). Compared with macrophages pre‐treated with ND MAT B cells, HFD MAT B cell pre‐treated macrophages significantly promoted mRNA expression of IL‐6 (*P* < 0.05) and TNF‐α (*P* < 0.01) and also inhibited adiponectin (*P* < 0.05) mRNA expression in adipocytes (Figure 4B,C). These results indicated that HFD MAT B cells might regulate adipocyte inflammation and endocrine function via macrophages.

### 3.5 B lymphocyte within inflamed MAT tended to migrate to the liver

**Figure 5:**
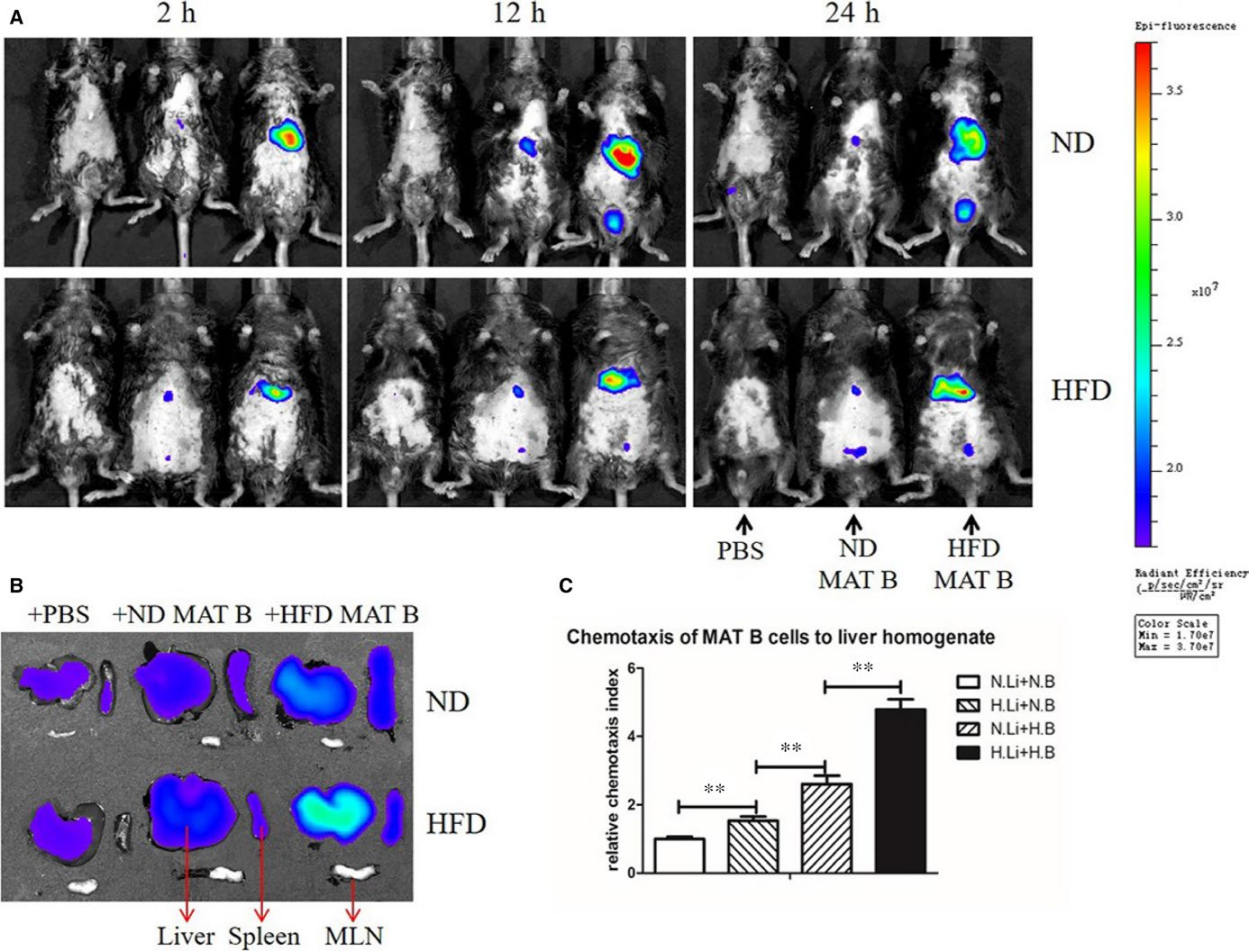
B lymphocyte within inflamed MAT tended to migrate to the liver. A, The near‐infrared scanning in vivo of mice receiving PBS, ND MAT B cells and HFD MAT B cells at three time points after adoptive transfer. B, Near‐infrared scanning of the liver, spleen and MLN of mice, after 24 h of receiving PBS, ND MAT B cells and HFD MAT B cells. C, Relative chemotaxis index of MAT B lymphocytes to liver homogenate. Values represent means ± SEM, **P < 0.01. H.B, MAT B cells from HFD mice; HFD, high‐fat diet; H.Li, liver homogenate from HFD mice; MAT, mesenteric adipose tissue; MLN, mesenteric lymph nodes; N.B, MAT B cells from ND mice; ND, normal diet; N.Li, liver homogenate from ND mice

To verify whether B lymphocytes in inflamed MAT could migrate to the liver, we used near‐infrared scanning in vivo and transwell chemotaxis assay in vitro. B cells isolated from MAT of HFD‐fed mice accumulated in the liver more than those from ND‐fed mice, whether the recipients were HFD‐ or ND‐fed mice (Figure 5A,B). Besides, B cells from HFD‐fed mice seemed to stay longer in the liver than those from ND‐fed mice, and this trend did not reduce at 24 hours after injection (Figure 5A,B). Compared with ND MAT B cells, a higher chemotaxis index (*P* < 0.01) of HFD MAT B cells to liver homogenates was observed, whether liver homogenates were made from ND or HFD‐fed mice (*P* < 0.01) (Figure 5C). The results of the transwell chemotaxis assay were consistent with near‐infrared scanning in vivo. All these results demonstrated that B lymphocytes in inflamed MAT tended to migrate to the liver.

### 3.6 B lymphocyte within inflamed MAT promoted inflammation of primary hepatocyte

**Figure 6:**
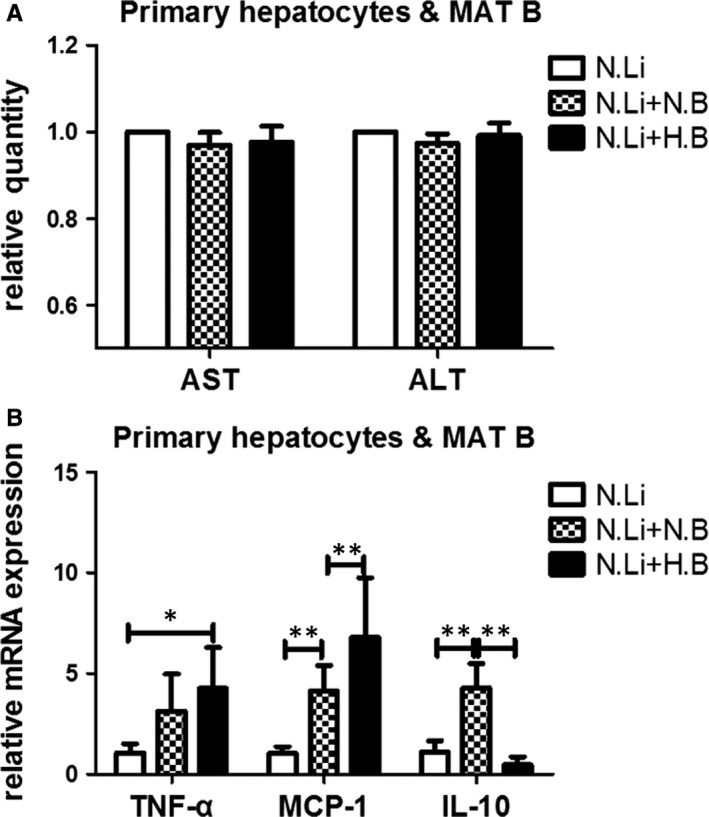
B lymphocyte within inflamed MAT promoted inflammation of primary hepatocyte. A, Aminotransferase of the culture supernatant in the co‐culture system of primary hepatocytes and MAT B cells, either from ND‐ or HFD‐fed mice. B, The relative mRNA expression of inflammatory cytokines in hepatocytes, specifically, TNF‐α, MCP‐1 and IL‐10. Values represent means ± SEM, *P < 0.05, **P < 0.01. HFD, high‐fat diet; IL, interleukin; MAT, mesenteric adipose tissue; ND, normal diet; TNF, tumour necrosis factor

To test whether B lymphocytes that migrated to the liver had effects on hepatocytes, we conducted a co‐culture experiment of MAT B lymphocytes and primary hepatocytes. B lymphocytes had no effects on the ALT or AST level in the cultural supernatant, neither B cells were isolated from ND‐fed nor HFD‐fed mice (Figure 6A). Compared to the control, ND MAT B cells promoted the mRNA expression of MCP‐1 (*P* < 0.01) and IL‐10 (*P* < 0.01) in hepatocytes (Figure 6B), and HFD MAT B cells elevated the mRNA expression of TNF‐α (*P* < 0.05) and MCP‐1 (*P* < 0.01) in hepatocytes. High‐fat diet MAT B cells induced higher expression of MCP‐1 (*P* < 0.01) and lower expression of IL‐10 (*P* < 0.01) in hepatocytes than those of ND MAT B cells (Figure 6B). These results indicated that increased MAT B lymphocytes that migrated to the liver promoted the inflammation of hepatocytes.

## 4 DISCUSSION

The theory of ‘multiple hits’ has been proposed to clarify the pathogenesis of NAFLD.^20^, ^21^ Mesenteric adipose tissue inflammation plays a pathogenic role in NAFLD. First, the incidence of NAFLD dramatically increases as MAT thickens,^22^, ^23^ and Bergman et  al suggest that MAT is a better predictor of insulin sensitivity than other depots of VAT.^7^ Next, in inflamed MAT, the cytokines and lipid decomposition products drained to portal vein are elevated, promoting the damage of liver.^5^, ^6^ Lastly, the inhibition of MAT inflammation can improve hepatic insulin resistance and hepatic steatosis.^10^ Nevertheless, the role of immune response within MAT is unknown. Here, we show that B lymphocytes infiltrate early in MAT and can promote the inflammation of adipocytes through macrophage‐mediated mechanisms. Besides, MAT B lymphocytes in HFD‐fed mice tend to migrate to the liver and promote the inflammation of hepatocytes.

The roles of macrophages and T lymphocytes in adipose tissue inflammation have been clarified in many studies,^24^, ^25^ as well the interaction between macrophages and T lymphocytes,^26^ but the role of B lymphocytes has not been adequately studied. In fact, the percentage of leucocytes in SVF of MAT is higher than that of EAT in mice, and B lymphocytes make up approximately 35% in SVF of MAT compared with little B cells in EAT.^27^ However, there is little research focusing on the infiltration order of lymphocytes in MAT. In EAT, B lymphocytes were found to be infiltrated early, followed by an increase in T cells and macrophages and finally by the appearance of insulin resistance in HFD‐fed mice.^28^ Winer et  al also evidenced that B cells accumulated in EAT as early as in 3 weeks of HFD feeding.^14^ In our study, the accumulation of B lymphocytes within MAT appeared earlier than macrophage infiltration. This highlighted the potential roles of B lymphocytes in the early stages of inflammation.

So far, it has been reported that B cells could promote tissue inflammation by producing cytokines, secreting immunoglobulin and modulating T cells.^14^, ^29^ Macrophage accumulation is supposed to be a marker of inflammation; new evidence suggests that B cells interact with macrophages. B cell deficiency results in the reduction of TNF‐α‐producing M1 macrophages in EAT,^14^ and transfer of EAT B2 cells from HFD‐fed wild‐type mice to HFD‐fed B^null^ mice leads to an increase in M1‐like macrophages.^15^ In vitro, EAT B cells from HFD‐fed mice promote expression of TNF‐α, IL‐6 and IL‐1β in M1 macrophages.^15^ Our results showed that MAT B lymphocytes isolated from HFD‐fed mice elevated the pro‐inflammatory cytokine secretion of macrophages and promoted their differentiation to type I macrophages.

Adipose tissue consists of various cells such as adipocytes, lymphocytes and fibrocytes. The causation role of B lymphocytes in MAT inflammation has not been evaluated yet. Fortunately, studies on B cells in EAT offer evidence to some extent. Winer et  al demonstrated that B cell accumulation promotes EAT inflammation and insulin resistance via modulation of T cells and production of IgG antibodies.^14^ B^null^ mice, lacking B lymphocytes, show improved insulin resistance, and adoptive transfer of EAT B2 cells into B^null^ recipients restores the effect of HFD to induce insulin resistance.^15^ DeFuria et  al found that B cells in blood of T2DM patients could promote inflammation through regulation of T‐cell function and an inflammatory cytokine profile; B^null^ mice with HFD showed attenuated inflammation of EAT, although hepatic steatosis was similar to that in normal HFD mice.^29^ We found that MAT inflammation alleviated and numbers of macrophages decreased in HFD‐fed B^null^ mice compared with HFD‐fed wild‐type mice. It was reported that B6.129 mice seemed to be less susceptible to HFD than C57BL mice.^30^, ^31^ Meanwhile, improved adipose tissue inflammation was also found in B cell depletion mice using anti‐CD20 antibody, compared with control mice.^14^ B cells secreted more inflammatory cytokines, regulated T cells function and produced pathogenic antibodies under the stimulation of HFD,^14^, ^29^ these have been demonstrated promoting adipose tissue inflammation. Thus, B cells were supposed to involve in MAT inflammation.

Among various cells in adipose tissue, adipocytes occupy maximum volume and possess great power of secreting cytokines. To our knowledge, there is no B cell receptor on the surface of adipocytes. It's well known that macrophages involve in adipocytes inflammation. Our results showed that macrophages, pre‐treated with MAT B lymphocytes, might induce adipocyte inflammation and decrease adiponectin expression. Meantime, decreased adiponectin was also supposed to promote adipose tissue inflammation.^32^ Thus, B lymphocytes might contribute to MAT inflammation partial through regulating macrophages inflammation and differentiation, and further studies about the mechanism of B cell and macrophage interaction were needed. These results indicate the non‐negligible role of B cells in adipose tissue inflammation.

B lymphocytes are recruited within the liver in NAFLD patients, in parallel with the worsening of parenchymal injury and lobular inflammation.^33^ In our previous study, we evidenced that intrahepatic B lymphocytes promoted NAFLD by secreting pro‐inflammatory cytokines, enhancing the activation of CD4^+^ T cells and their differentiation into Th1 cells.^34^ Apart from intrahepatic B cells, the effects of B cells from circulation or EAT on metabolic syndrome have also been described in several studies,^35^, ^36^ but whether B cells from spleen or EAT can migrate to the liver is unknown. Here, we found that early infiltration of B cells occurred both in the liver and MAT during the development of NAFLD; thus, we speculated that MAT B cells could migrate to the liver and participate in liver injury. In fact, the cycle of immune cells among various organs is the fundamental guarantee of a normal immune response. The capability of gut‐ and blood‐derived B cells to migrate to the liver has been demonstrated, and this phenomenon promotes the development of NAFLD.^18^, ^37^ Using adoptive transfer of MAT B cells and near‐infrared scanning in vivo as well as transwell assay, we showed that B cells within inflamed MAT that migrated to the liver increased, promoting the inflammation of hepatocytes. Although B cells within MAT of ND‐fed mice also promoted the hepatocytes to secrete cytokines, near‐infrared scanning in vivo showed few B cells migrating to livers in ND‐fed mice.

**Figure 7:**
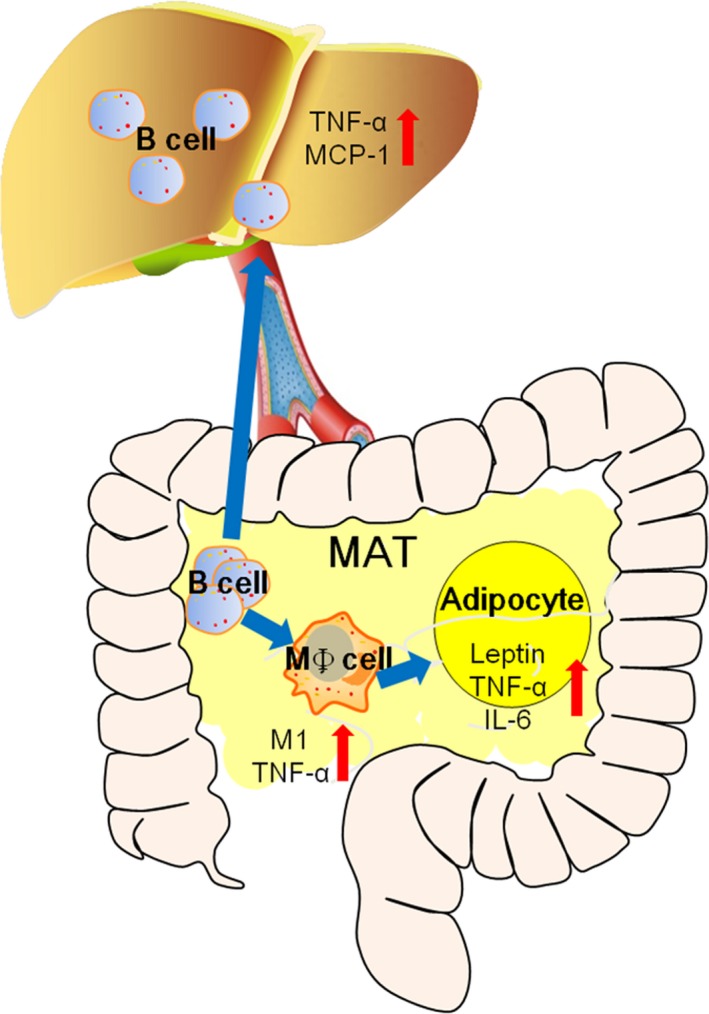
Schematic model for the role of MAT B cell in local inflammation and hepatic inflammation. B lymphocytes increased early in MAT of HFD‐fed mice and promoted macrophage differentiation into type I macrophages as well as secretion of more pro‐inflammatory cytokines. The effects of B lymphocytes on macrophages resulted in MAT inflammation. Besides, B lymphocytes within MAT could migrate to the liver and promote hepatic inflammation. HFD, high‐fat diet; MAT, mesenteric adipose tissue

In this study, we showed that B lymphocytes within MAT participated in NAFLD not only by partly promoting MAT inflammation by regulating macrophages, but also by migrating to the liver and inducing the inflammation of hepatocytes (Figure 7). Considering the early infiltration of B lymphocytes in longitudinal development of NAFLD, we believe that B lymphocytes may play a more important role than that already elucidated. All these results highlight the importance of B cells in the communication between the immune system and inflammation as well as MAT and liver dialogue.

## CONFLICT OF INTEREST

None.
