# Supplementary material for: Mesenteric adipose tissue B lymphocytes promote local and hepatic inflammation in non‐alcoholic fatty liver disease mice
Source: J Cell Mol Med. 2019 Feb 17;23(5):3375–85. doi: 10.1111/jcmm.14232 (PMC6484337; doi:10.1111/jcmm.14232)
Supplement: Supplementary file 1 [file JCMM-23-3375-s001.docx]

**Supplemental Table 1. SYBR Green PCR Primer sequences of target genes in mice**

| Primers | Forward 5’ →3’ | Reverse 5’ →3’ |
| --- | --- | --- |
| GAPDH | TCAACAGCAACTCCCACTCTTCCA | ACCCTGTTGCTGTAGCCGTATTCA  GGCAGCCTTGTCCCTTGA CCAGCCTACTTGGGATCA TCAAATTCATTCATGGCCTTGT  TATAAGCGGCTTCTCCAGGCT  TGGCTATCTGCAGCACATTTTG  CCATTGCACAACTCTTTTCTCATTC GCAAGGAGGACAGAGTTTATCGTG |
| TNF-a  MCP-1  IL-10  Adiponectin  Leptin  IL-6  F4/80 | CAGCCGATGGGTTGTACCTT GCAGTTAACGCCCCACTCA TGGAGCAGGTGAAGAGTGATTTT  TGGAATGACAGGAGCTGAAGG  GGGCTTCACCCCATTCTGA  AAGTCGGAGGCTTAATTACACATGT  CTTTGGCTATGGGCTTCCAGTC |  |
